# Tense bullae in a pediatric patient

**DOI:** 10.1016/j.jdcr.2025.08.032

**Published:** 2025-09-18

**Authors:** Iman Ali, Travis S. Dowdle, Gabriela Villa, Frank T. Winsett, Brandon P. Goodwin

**Affiliations:** a Department of Internal Medicine, Texas Health Presbyterian Dallas, Dallas, Texas; b Department of Dermatology, University of Texas Medical Branch, Galveston, Texas; c School of Medicine, University of Texas Medical Branch, Galveston, Texas

**Keywords:** autoimmune, bullae, chronic bullous dermatosis of childhood, dapsone, direct immunofluorescence, hematoxylin and eosin, linear IgA bullous disease, pediatrics

## Case description

**Fig 1:**
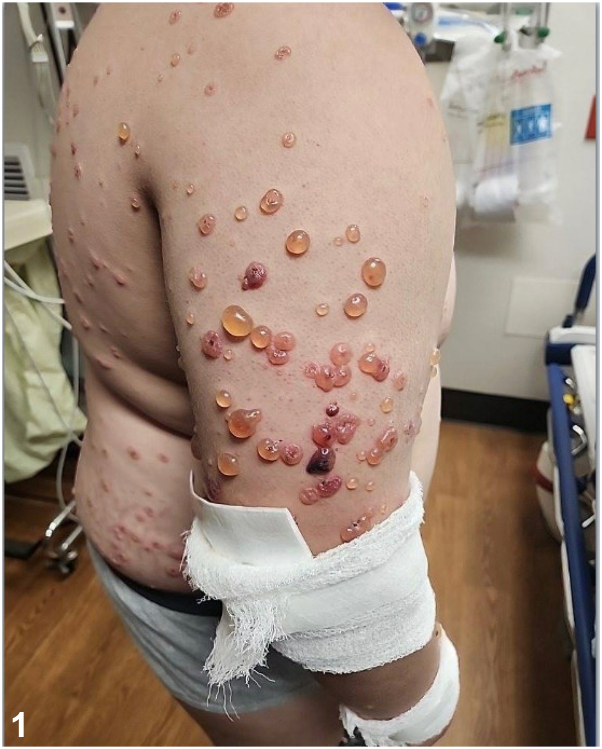

A 10-year-old previously healthy male presented with a 5-day history of itchy, tense bullae to arms, trunk, and face. New blisters can be seen at the periphery of older lesions forming annular and arciform configurations with central crusting (Fig 1). He had no recent history of infections or exposure to medications, including antibiotics or nonsteroidal anti-inflammatory drugs.

**Fig 2:**
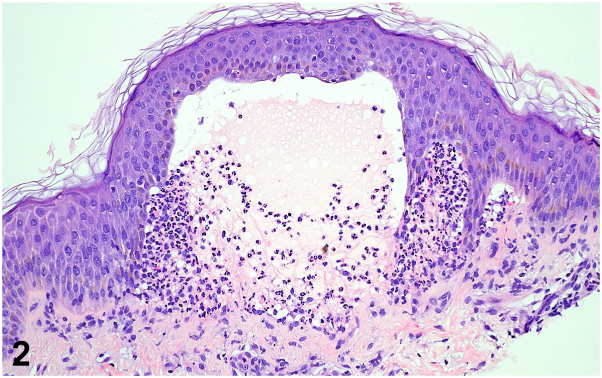

**Fig 3:**
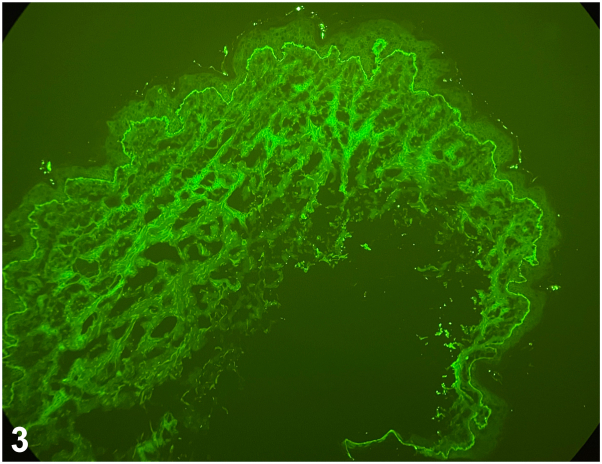

Skin biopsy was obtained for hematoxylin and eosin and direct immunofluorescence (DIF). Hematoxylin and eosin revealed subepidermal bullae with abundant neutrophils (Fig 2), and DIF is shown below (Fig 3). G6PD screening was normal.

**Fig 4:**
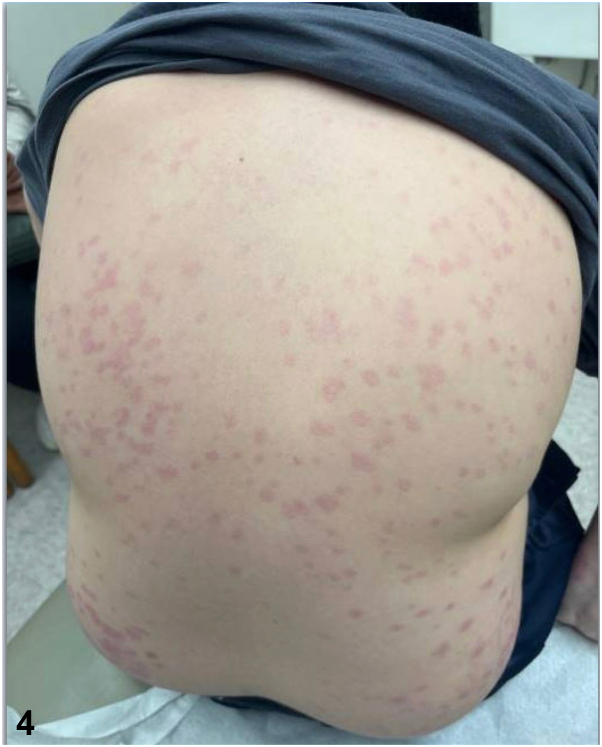

The patient was started on dapsone 50 mg daily. At the 4-week follow-up, bullae and pruritus had completely resolved, with only postinflammatory erythema remaining (Fig 4).

**Question 1: What is the most likely diagnosis?**

A. Bullous pemphigoid
B. Chronic bullous dermatosis of childhood
C. Dermatitis herpetiformis
D. Epidermolysis bullosa acquisita
E. Pemphigus vulgaris

**Answers:**

A. Bullous pemphigoid – Incorrect. Bullous pemphigoid presents with large, tense blisters that may be hemorrhagic. This condition typically affects older adults, not children. While histology may show subepidermal bullae, the inflammatory infiltrate is usually eosinophilic, not neutrophilic. Additionally, DIF shows linear IgG and C3 rather than IgA along the basement membrane zone. Systemic corticosteroids are considered first-line treatment.^1^
B. Chronic bullous dermatosis of childhood – Correct. CBDC, also known as linear IgA bullous dermatosis, is the most common acquired blistering disorder in children. Patients present with tense bullae in annular or arciform patterns often described as a “cluster of jewels.” Histology reveals subepidermal bullae with neutrophilic infiltrate, and DIF shows linear IgA deposition at the basement membrane. This condition responds rapidly to dapsone further supporting the diagnosis of CBDC.^1^
C. Dermatitis herpetiformis – Incorrect. Dermatitis herpetiformis is characterized by grouped vesicles on the extensor surfaces (elbows, knees, buttocks) and is strongly associated with celiac disease. DIF shows granular IgA deposition in the dermal papillae, not linear deposition. A gluten-free diet and dapsone are effective treatment options.^1^
D. Epidermolysis bullosa acquisita – Incorrect. Epidermolysis bullosa acquisita is a chronic blistering disease and is very rare in pediatric populations. This condition presents with trauma-induced bullae, healing with scarring or milia. Autoantibodies in epidermolysis bullosa acquisita target type VII collagen that is part of the anchoring fibrils of the epidermal basement membrane. DIF shows linear IgG and C3 at the basement membrane. Combination therapy with prednisone and dapsone is effective in controlling this disease.^1^
E. Pemphigus vulgaris – Incorrect. Pemphigus vulgaris is characterized by flaccid bullae that quickly rupture, leaving painful and persistent erosions and crusts on the skin. This condition is commonly associated with mucosal involvement. DIF reveals intercellular IgG deposition in a “chicken wire” pattern which is not seen in this case. Systemic corticosteroids are considered mainstay therapy.^1^

**Question 2: Which of the following DIF findings seen in** Fig 3 **is most characteristic of the suspected diagnosis?**

A. Granular IgA deposits in the dermal papillae
B. Linear IgG and C3 deposition at the basement membrane zone
C. Linear IgA deposition along the dermal-epidermal junction
D. Intercellular IgG throughout the epidermis
E. Lack of antibodies detected on the basement membrane

### Answer discussion

The correct answer to question #2 is C. Linear IgA deposition along the dermal-epidermal junction. The patient’s presentation of tense bullae arranged in a “cluster of jewels” is most consistent with CBDC, the pediatric variant of linear IgA bullous dermatosis.^2^ This autoimmune blistering disorder typically affects children under the age of 5, although older onset – as in this 10-year-old – is possible. Lesions often localize to the lower abdomen, groin, thighs, and periorificial areas, including the eyes and mouth.^2^

On histology, CBDC demonstrates subepidermal bullae with a predominately neutrophilic infiltrate. However, the hallmark diagnostic feature is seen on DIF which reveals linear deposition of IgA along the dermal-epidermal junction, distinguishing CBDC from other immunobullous diseases.^3^

The pathogenesis involves IgA autoantibodies targeting components of the basement membrane zone, particularly the 120-kDa fragment of BP180/collagen XVII and its cleavage product, the 97-kDa fragment.^2^ While the precise trigger is often unclear, associations have been reported with medications (such as vancomycin, NSAIDs, and antibiotics), infections, autoimmune conditions, and gluten-sensitive enteropathies.^2^

First-line treatment for CBDC is dapsone, which alters neutrophil function through several mechanisms including inhibition of chemotaxis, oxidative burst, and myeloperoxidase activity thereby reducing inflammation and subepidermal blister formation. Improvement is rapid, typically within days. Pediatric dosing starts at 0.5 mg/kg/d and may be increased to a maximum of 2 mg/kg/d based on clinical response.^4^

Regular monitoring during dapsone is essential and should include complete blood counts, reticulocyte counts, liver function tests, and methemoglobin levels.^5^ This should be done in patients even with normal G6PD activity. Serious adverse effects include dapsone hypersensitivity syndrome, a life-threatening condition which can result in end organ failure.^5^ Other side effects include hemolysis, gastrointestinal upset, and hepatotoxicity. In treatment-refractory cases, adjective therapies such as systemic corticosteroids, colchicine, penicillin-class antibiotics, or immunosuppressants (eg, cyclosporine) may be considered.^2^

The prognosis for CBDC is generally favorable with most children achieving spontaneous remission by puberty. However, recurrence or persistence into adulthood is possible. Ongoing dermatologic follow-up is essential to monitor both disease activity and treatment safety.

## Conflicts of interest

None disclosed.
